# The parallel mediating role of time and financial resources in achievement motivation and non-credential learning

**DOI:** 10.3389/fpsyg.2025.1579281

**Published:** 2025-08-04

**Authors:** Yi Cao, Yi Tian, Yuan Hu, Juan Kou

**Affiliations:** ^1^Academic Affairs Office of Sichuan Normal University, Chengdu, China; ^2^Institute of Brain and Psychological Science, Sichuan Normal University, Chengdu, China

**Keywords:** non-degree credentials courses, achievement motivation, self-directed learning time, financial resources, parallel mediation

## Abstract

**Introduction:**

The surge in non-degree credentials, such as micro-credentials and MOOC, in China is being driven by evolving labor market needs and the growing demand for specialized skills. Participation in such programs is influenced by various internal factors, particularly achievement motivation, as well as external factors like time management and financial resources. Yet, the impact of these factors on willingness to participate remains unexplored.

**Methods:**

This study examines the relationship between achievement motivation and engagement in non-degree credential education, focusing on the mediating roles of self-directed learning time and financial capability. Data from 3,397 Chinese college students aged 18-22 were analyzed using a parallel mediation model. Structural equation modeling was employed to analysis the data.

**Results:**

The findings reveal that both learning time and financial resources significantly mediate this relationship, each with distinct levels of influence. Students with higher achievement motivation tend to invest more time and money in non-degree credential learning, thereby enhancing their likelihood of participation.

**Discussion:**

These results highlight the critical roles of time management and financial capacity in shaping students’ involvement in non-degree credentials, offering valuable insights for educational policymakers and institutions seeking to enhance accessibility and support for non-degree credential programs in higher education.

## 1 Introduction

In the last decade, there has been a notable surge in non-degree credentials (e.g., Micro-credential course, massive open online courses) (National Center for Education Statistics, 2024) in the world, and also the pursuit of non-degree credentials has been increasingly embraced by higher education institutions in China (Gallagher, 2022). This trend is influenced by various factors, such as the escalating costs of conventional higher education, shifts in the job market, and the escalating demand for specialized skills and knowledge in the workforce. As the education landscape evolves in response to the growing demands, students are increasingly turning to non-credential learning opportunities to enhance their academic portfolios and professional skill. In addition to providing a competitive edge in employment, non-credential courses also offer students avenues for personal development and intellectual enrichment, helping them stay adaptable in a rapidly changing global economy. Non-credential learning refers to structured or intentional learning activities that do not lead to formal certification, such as auditing MOOCs or workplace training without awards. This definition is consistent with the OECD’s framework, which emphasizes learning outcomes over the setting (Werquin, 2010). Compared with normal learning in university, non-credential learning, especially micro-credentials, aims to provide students with spare capacity to focus on a group of core courses in an emerging field. Non-credential learning (e.g., Micro-credentials) are seen as a way of advancing career and providing a skilled workforce for rapidly changing industries for individuals (Desmarchelier and Cary, 2022; Oliver, 2019). In university, micro-credential learning can broaden students’ channels of interdisciplinary learning, meet the needs of cultivating compound talents and students’ personalized development. In contrast to informal and self-directed learning, non-credential learning operates within formalized frameworks comprising set timelines and prescribed course arrangements.

However, the factors influencing student participation in non-degree credentials courses are complex. While external conditions such as the availability of resources and institutional support play an essential role, internal motivations, particularly achievement motivation, are key determinants of student engagement in non-credential learning activities (Yilik, 2021; Van der Hijden and Martin, 2023; Pirkkalainen et al., 2023). Achievement motivation refers to an individual’s desire to achieve success and reach a high level of accomplishment in their academic or career-related pursuits (Linnenbrink-Garcia and Maehr, 2011). For many students, this intrinsic motivation drives them to seek out additional learning experiences that go beyond the requirements of their degree programs, including non-credential courses (Friðriksdóttir, 2021). Questionnaires such as the Achievement Motives Scale, AMS (Heckhausen, 1991; Rheinberg, 2004) are employed for the assessment of self-attributed motives. In the context of non-credential learning, achievement motivation is particularly significant because these courses often require self-directed effort and financial investment. Unlike formal coursework that is typically required as part of a degree program, non-credential learning relies heavily on the student’s personal desire to improve their knowledge or skills. Students who exhibit high levels of achievement motivation are more likely to take advantage of these opportunities to enhance their professional qualifications or pursue personal interests.

While internal motivation is crucial, external resources-such as time and financial capacity-also significantly shape students’ ability to participate in non-credential courses. The commitment of self-directed learning time is essential for successfully engaging in these activities. Students who can effectively manage their time and allocate sufficient hours to self-directed study are more likely to participate in non-credential courses. Time management, therefore, becomes a key factor in facilitating or constraining participation in such learning opportunities (Wolters and Brady, 2021).

Financial resources are another critical factor influencing participation. While many non-credential programs offer valuable learning experiences, they often require financial investment, either in the form of tuition fees or the cost of course materials (Yang, 2023; Oliver, 2021). Students with access to financial resources are more likely to invest in these learning opportunities. However, financial constraints can act as a significant barrier for students, limiting their ability to participate, regardless of their motivation. Consequently, understanding how financial resources mediate the relationship between achievement motivation and engagement in non-credential learning activities is crucial for promoting equitable access to education.

This study aims to examine the roles of two external resources (self-directed learning time and financial resources) on the relationship between achievement motivation and participation in non-credential learning activities. By exploring these dynamics, the research aims to shed light on the ways in which both internal and external factors converge to influence students’ willingness and ability to engage in non-credential learning courses. Previous research has shown that self-directed time and self-directed financial resources are distinct investment dimensions in academic settings (Macan et al., 1990). Time management skills and financial capacity are rarely correlated among traditional students (Credé and Kuncel, 2008). We propose treating two factors as parallel mediators. Given the increasing importance of non-credential learning in today’s educational and professional landscapes, understanding these predictors can help educational institutions develop targeted strategies to support student engagement, optimize learning outcomes, and ensure that students from diverse backgrounds have equal opportunities to participate.

Through this investigation, the study contributes to the broader conversation about how to foster achievement motivation and create supportive learning environments that accommodate the diverse needs and circumstances of students. In doing so, it offers valuable insights for educators, policymakers, and administrators seeking to enhance the accessibility and effectiveness of non-credential learning opportunities in China’s higher education system. Additionally, the findings of this study have the potential to inform global discussions on lifelong learning, personal development, and the future of education in an increasingly knowledge-driven society.

## 2 Methods

### 2.1 Participants

A sample of 3,397 participants (age range 18–23) was recruited from one university from local China in Sichuan (Sichuan Normal University). As is typical for this kind of school, the sample comprised less males (*N* = 753; 22.17%) than females (*N* = 2,644; 77.83%). Our sampling strategy ensured broad representational of the undergraduate population across four academic years and four majors. Identify the five majors we want to study (e.g., STEM, Humanities, Business, Arts), and then consider the need for undergraduates in sampling according to previous study (Pascarella and Terenzini, 2005; Hutchison, 2017). Utilize convenience sampling to sample from available students in each major, by collaborating with department heads to recruit students, satisfying inclusion criteria of current enrollment (18–25 years) and voluntary participation. The final sample (*N* = 3,397) comprised STEM (58%, *n* = 1970), Humanities (18.5%, *n* = 629), Education (10.6%, *n* = 360), Business (9.6%, *n* = 325) and Arts (3.3%, *n* = 113). Furthermore, the final sample (*N* = 3,397) comprised Year 1 (6.5%, *n* = 222), Year 2 (17.9%, *n* = 610), Year 3 (23.4%, *n* = 798), and Year 4 (52.0%, *n* = 1,767) students from five different majors. We verify that the study is in accordance with established ethical guidelines. Approval by the local ethics committee was conducted. Participation was voluntarily. Before testing, we received informed consent forms from the students.

### 2.2 Measurements

#### 2.2.1 Self-achievement motivation (SAM)

Self-achievement motivation is measured by the Achievement Motivation Scale (AMS) (Ye and Hagtvet, 1992), which is a widely used instrument of the achievement motivation. This scale assesses two dimensions: the desire for success and fear of failure. The Chinese version of AMS was psychometrically sound and could be applied in China (Zhang et al., 2016). Scores are calculated using a 4-point Likert scale, with higher scores indicating stronger achievement motivation in individuals. The scale demonstrated good reliability with a Cronbach’s alpha coefficient of 0.913, and alpha coefficients of 0.922 and 0.936 for the two dimensions, respectively in current sample. The AMS consists of 30 items, with 15 items each for the pursuit of success and avoidance of failure motivations. The AMS is user-friendly as researchers can easily collect data by distributing the questionnaire to participants for self-completion. The scale exhibits high reliability and validity, with coefficients exceeding 0.8, enabling the assessment of individual personalities and factors contributing to occupational and professional success.

#### 2.2.2 Self-directed learning time

Self-directed learning time was assessed using a single item rated on a 4-point Likert scale, ranging from 1 (2 h and below) to 4 (8 h and above) per week. Participants were asked, “With the current study schedule, how much time can you dedicate to your self-directed learning courses?”

#### 2.2.3 Available money for tuition

Available money for tuition was measured by a single item asking “Within what range of fees per single credit for extra course are you available to pay?,” which was rated on a 4-point Likert scale, ranging from 1 (50–100 RMB) to 4 (above 200 RMB) per credit.

#### 2.2.4 Willingness for extra non-credential courses

The willingness to enroll in additional courses was evaluated through a single item on a 5-point Likert scale, with responses ranging from 1 (slight) to 5 (highly) inclined to participate. Participants were asked, “If a noncredit course program (e.g., micro-credential program) were to be introduced by the school, would you be inclined to register for it, commit to completing the program, and obtain the certificate upon finishing?”

### 2.3 Data analysis

All incomplete responses and invalid entries (e.g., duplicate responses) were excluded were systematically excluded during data cleaning. Eliminated identical IP and students number addresses and timestamps. To examine the correlation between Willingness to participate in non-credential courses (WEC), Self-achievement motivation (SAM), Available money for tuition (AMT), and Self-directed learning time (SDT), Pearson correlations were performed with Bonferroni correction for multiple comparisons. In order to analyze our research model and determine the parameter estimates, we utilized Structural Equation Modeling (SEM) with Robust Maximum Likelihood estimation, using Mplus software (Muthen and Muthén, 1998) (version 8.3). The independent variables in our model (self-directed learning time and available money for tuition) were treated as observed variables. The mediators (self-directed learning time and available money for tuition) and the outcome (willingness to take extra courses) were considered variables for each measurement. Since indirect effects do not adhere to a normal distribution (MacKinnon et al., 2002), we tested the hypothesized indirect effects using bootstrapping methods with 10,000 replications and 95% Confidence Intervals (CI).

## 3 Results

### 3.1 Correlation

The results presented above for the analysis of correlation of Willingness to participate extra non-credential courses (WEC), Self-achievement motivation (SAM), Available money for tuition (AMT) and Self-directed learning time (SDT) were significant. Firstly, as shown in Table 1, SAM (*r* = 0.205, *p*_Bonferroni_ ≤ 0.001) was found to be significantly correlated with WEC. The result indicated AMT (*r* = 0.180, *p*_Bonferroni_ ≤ 0.001) as well as SDT (*r* = 0.153, *p*_Bonferroni_ ≤ 0.001) were also correlated with WEC. The detailed correlation is shown in Table 1. All *p*-values from the correlations that were converted to *p*_Bonferroni_-values with the code *p*.adjust in R for correcting the correlations for multiple comparisons.

**Table 1:** Correlation between Willingness to participate extra non-credential courses (WEC), Self-achievement motivation (SAM), Available money for tuition (AMT) and Self-directed learning time (SDT).

Factors	1	2	3	4
WEC	1			
SAM	0.205^**^	1		
AMT	0.180^**^	0.079^**^	1	
SDT	0.153^**^	0.136^**^	0.055^**^	1

*p < 0.05, **p < 0.01.

### 3.2 Mediation analyses

**Figure 1:**
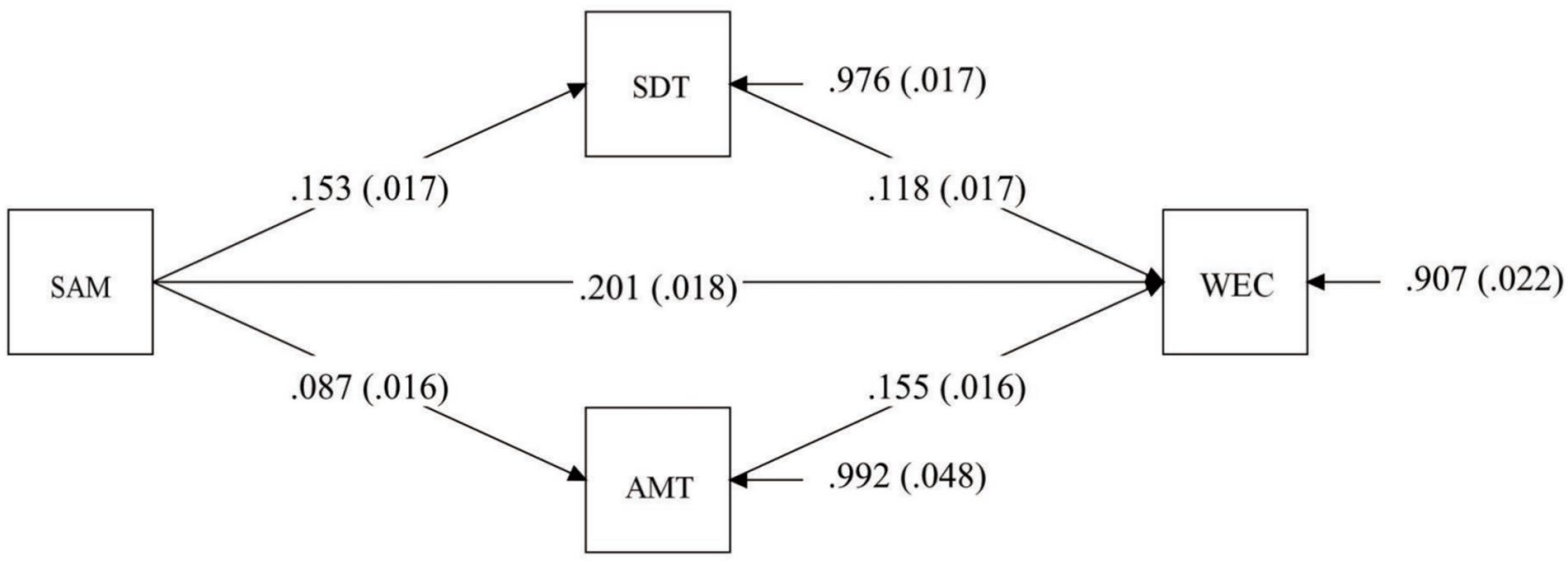
Parameter estimates for the hypothesized research model. Coefffcients are standardized. Standard errors are in brackets.

We implemented the use of 10,000 bootstrapping samples (resamples) to ascertain the confidence intervals and significance of the indirect effects. We investigated whether Learning Resources (Self-directed learning time and Available money for tuition) mediated the relationship between Self-achievement motivation and Willingness for extra courses. The hypothesized research model showed a good fit to data (*χ*^2^(1) = 5.00, *p* < 0.026; CFI = 0.99, TLI = 0.95, RMSEA = 0.03, SRMR = 0.01). Next, the standardized regression coefficients (betas) for direct and indirect effects are reported in Table 2. As expected, the coefficients for the direct effects showed that Self-achievement motivation was positively associated with Willingness for extra courses and positively associated with Learning Resources (Self-directed learning time and Available money for tuition) (details see Figure 1). The unstandardized regression coefficients for direct and indirect effects are also reported for illustration purposes in SI for each sample.

**Table 2:** Estimates for the hypothesized indirect effects and effect sizes.

Independent measure (IV)	Total effect			Direct effect			IV-M			M-DV			Indirect effect		
	*c*			*c*’			*a*			*b*			a*b		
	*β*	SE	95% CI	*β*	SE	95% CI	*β*	SE	95% CI	*β*	SE	95% CI	*β*	SE	95% CI
Self-achievement motivation (SAM)	0.232	0.018	[0.197, 0.267]	0.201	0.018	[0.166, 0.235]	0.153	0.017	[0.119, 0.186]	0.118	0.017	[0.085, 0.152]	0.018	0.003	[0.012, 0.025]
Self-achievement motivation (SAM)							0.087	0.016	[0.057, 0.119]	0.155	0.017	[0.123, 0.187]	0.014	0.003	[0.008, 0.020]

CI, Confidence Interval; SE, standard error.

We implemented the use of 10,000 bootstrapping samples (resamples) to ascertain the confidence intervals and significance of the indirect effects (see Table 2). The results showed that the indirect effects of Self-achievement motivation on Willingness for extra courses by Learning Resources (Self-directed learning time and Available money for tuition) were significant. An inspection of the explained variance in each of the models showed that altogether, Self-achievement motivation and Learning Resources (Self-directed learning time and Available money for tuition) accounted for 9.2% of the variability in Willingness for extra courses.

## 4 Discussion

The current study investigated the association between achievement motivation and willingness of engagement in non-credential learning activities by exploring the mediating roles of two other resources, self-directed learning time and financial resources willing to afford, which would be influenced by internal motivation to some extend. The results offer valuable insights into how these factors interact to impact student involvement in alternative educational pursuits. The findings confirm the significance of achievement motivation as a key determinant of participation in non-credential learning activities. Individuals with higher levels of achievement motivation demonstrate a greater propensity to participate in such activities, indicating a direct link between motivation and the pursuit of additional learning opportunities outside formal education settings. The analysis demonstrated that self-directed learning time plays a significant mediating role in the relationship between achievement motivation and engagement in non-credential courses. Motivated students are more likely to dedicate their time to non-credential learning activities. Additionally, financial resources were identified as another significant mediator. Students with heightened achievement motivation tend to invest more available financial resources in non-credential learning pursuits, underscoring the importance of financial capacity in facilitating such learning opportunities. Furthermore, no significant difference of mediation was observed in the allocation of self-directed learning time versus financial resources.

### 4.1 Self achievement motivation and non-credential learning participation

The results confirm that achievement motivation is a crucial determinant of participation in learning activities especially non-credential learning activities. Our findings indicate that individuals with higher levels of achievement motivation are more likely to engage in non-credential learning opportunities. This aligns with existing literature on achievement motivation, reinforcing its role in driving educational engagement (Vogt et al., 2021; Dwijuliani et al., 2021). Motivation is crucial for successful curriculum implementation. Learners’ motivation is key to achieving educational goals and plays a significant role in the teaching-learning process. Teachers must inspire and sustain intrinsic motivation in students for effective learning (Filgona et al., 2020). In non-credential learning, students are primarily differentiated by their motivation to self-improve or acquire knowledge for personal growth more. Regardless of the specific source from students’ perspective, this motivation is what drives non-credential learners to actively seek out and engage in learning opportunities. It is this intrinsic drive that sets them apart from traditional students and allows them to approach their education with a unique perspective and determination. Additionally, this motivation often translates into a deeper understanding and retention of the material, as non-credential learners are driven by a genuine interest in the subject matter rather than simply meeting academic requirements. As a result, non-credential learning can be a highly rewarding and fulfilling experience for those who are truly motivated to learn and grow.

Furthermore, current study extends the understanding of this motivation by highlighting its specific impact on non-credential learning participation, which has been under-explored in previous research.

### 4.2 Mediating role of self-directed learning time and financial resources

For the self-directed learning time significantly mediates the relationship between achievement motivation and participation in non-credential courses. Students who are motivated to achieve are more likely to allocate their time towards non-credential learning activities. This finding underscores the importance of time management skills in self-directed learning environments.

Upon transitioning from senior high school to university, students commonly encounter heightened levels of autonomy and responsibility, as they are compelled to partake in a broader spectrum of educational pursuits beyond traditional class hours, autonomously and under self-regulation (Banahan and Mullendore, 2014). Researchers on the connections between time management and students’ engagement, learning indicated that time management is a key self-regulatory process for students to actively manage their academic activities (Wolters and Brady, 2021). Enhanced self-achievement motivation can lead to improved management development, enabling individuals to allocate more self-directed time to their interests and participation in learning activities. In all, it may suggests that institutions should provide support structures that help students manage their time effectively, thereby maximizing the benefit of their achievement motivation.

Financial resources also emerged as a significant mediator. In this study, we investigated the mediating function of family income in the association between self-achievement motivation and the inclination to engage in non-credential learning. Despite the absence of a notable mediating influence, a propensity to allocate funds towards tuition was noted. This underscores its connection to students’ inclination to commit resources to supplementary non-credential programs, albeit without a direct impact from economic income. Students with higher achievement motivation tend to invest more financial resources in non-credential learning activities, indicating that the ability to allocate financial resources is a key factor in facilitating these learning opportunities. In previous study, the socioeconomic status and cultural capital of a family significantly influence students’ academic achievements and participation in extracurricular activities (Yang, 2023). Furthermore, our currents results showed that time is a stronger mediator than financial resources between the relationship of Achievement Motivation and Non-Credential Learning willingness which is consistent with the resource scarcity principle in educational psychology in students sample (Hobfoll, 1989). The dominance of time mediation aligns with the resource scarcity principle in educational psychology (Hobfoll, 1989). Student workload has been recognized as a major factor in the teaching and learning environment (Kyndt et al., 2014). University students face fragmented schedules due to academic overload and social commitments, making discretionary time scarcer than financial resources. Notably, micro-certificate programs allow flexible funding through part-time work or family support, whereas time investment implies irreversible opportunity costs (Shah et al., 2012). Over the last two decades, most people report feeling persistently ‘time poor’ as if they have too many things to do and not enough time to do them. Time poverty is linked to lower well-being, physical health and productivity (Giurge et al., 2020). Research shows ‘time accessibility’ and financial subsidies are important in promoting skill acquisition (Tripon, 2022), so, micro-certificate fees can be flexibly managed through part-time work when these students have time and willingness to learn it.

Current study extends previous research by exploring the willingness of students to invest in non-credit supplementary courses relative to their family income. It not only illuminates a crucial barrier to participation for financially constrained students but also underscores the subjective motivation tied to students’ aspirations for personal accomplishment. To enhance equitable access to non-credential learning, educational institutions and policymakers should not only focus on financial support mechanisms, such as scholarships, grants, or subsidized programs but also integrate strategies that promote intrinsic motivation factors.

### 4.3 Implications for educational policy and practice facilitating achievement motivation

Our findings suggest that fostering achievement motivation could enhance students’ willingness to engage in non-credit courses. Given the pivotal role of achievement motivation, educational programs should incorporate strategies to cultivate this trait in students. For instance, interventions could focus on enhancing students’ beliefs in their competencies, utilizing mindset interventions to shift students’ attitudes from fixed to growth mindsets regarding their capabilities, and emphasizing the practical value of the content being learned (Wigfield et al., 2021). Additionally, initiatives such as mentorship programs, goal-setting workshops, and integrating motivational components into the curriculum may also be beneficial. By actively promoting achievement motivation, we can create a more engaged and motivated student body, leading to increased participation in non-credential courses.

#### 4.3.1 Facilitating students to allocate more time for self-directed learning

Educational policies and institutional practices should aim to cultivate environments that support self-directed learning. This could include providing resources and tools for effective time management, offering flexible learning schedules, and creating dedicated spaces for non-credential learning activities on campus. Those who define goals, objectives, and priorities play a key role in managing time efficiently, resulting in enhanced time management performance (Alvarez Sainz et al., 2019). To enhance time management skills, students are advised to utilize a diary for goal setting and employ the time-boxing technique to allocate specific time frames for completing tasks. This approach facilitates prioritization and decision-making. When defending a dissertation or presenting assignments in seminars, students should demonstrate their time-boxing strategies and articulate any adjustments made from the original plan to accommodate the task at hand. It is crucial for students to cultivate an awareness of their time utilization through tracking, fostering flexibility, and the ability to decline non-essential commitments to mitigate procrastination and distractions (Alvarez Sainz et al., 2019). Educational institutions should offer workshops or courses on time management to further support students in developing this essential skill.

#### 4.3.2 Financial support and access

Addressing financial barriers to non-credential learning is crucial to ensuring that all students, regardless of their financial background, have the opportunity to engage in valuable learning experiences, especially those with high achievement motivation but limited financial support. Institutions should consider forming partnerships with external organizations to secure funding and create affordable learning options.

### 4.4 Limitations

While the study provides robust insights, it is based on one colleague data set from college students in China, and cultural factors may influence the generalizability of the findings to other contexts. Future research should consider cross-cultural studies to validate the applicability of these results in different educational settings. Additionally, employing a longitudinal study design could yield more profound insights into the influence of achievement motivation and its mediators on participation over an extended period. Subsequent studies could delve into the lasting effects of achievement motivation on career outcomes and lifelong learning.

Future research could explore the long-term impact of achievement motivation on career outcomes and lifelong learning. Future studies could also investigate other potential mediators such as peer influence, institutional support, and digital literacy, which might further elucidate the complex dynamics of non-credential learning engagement.

## 5 Conclusion

The findings from this study underscore the dual importance of time and financial resources in shaping students’ involvement in non-credential learning activities. By identifying achievement motivation as a key driver and highlighting the mediating roles of learning time and financial investment, this research offers valuable insights for educational policy and practice. To foster a more inclusive and supportive learning environment, educational institutions and policymakers must address the barriers to non-credential learning and leverage the motivational attributes of their students effectively.

The level of students’ achievement motivation will ultimately impact the sustainability and effectiveness of offering micro-credentials. For the students surveyed, possessing awareness of micro-credentials is essential. This entails having personal achievement motivation and committing sustained time and effort to learning, leading to a deep sense of identity, satisfaction, and accomplishment in micro-credentialing. The higher the motivation for success, the more enduring the engagement in micro-credential learning, and the more willing individuals are to utilize high-quality resources such as curriculum systems, faculty teams, and interdisciplinary programs for self-improvement, ultimately achieving personal success and self-fulfillment. Therefore, innovative talent development models and reform initiatives implemented by local universities often garner greater emotional support and ideal realization among learners with higher achievement motivation. This, in turn, results in higher levels of engagement and enthusiasm in learning, making micro-credential programs and other educational reforms more stable and enduring.

## Data Availability

The datasets presented in this article are not readily available because premature release could compromise the integrity of our ongoing study/findings. Requests to access the datasets should be directed to the first corresponding author (caoyi0416@126.com).
